# Study of the Tissue Distribution of TLQP-21 in Mice Using [^18^F]JMV5763, a Radiolabeled Analog Prepared via [^18^F]Aluminum Fluoride Chelation Chemistry

**DOI:** 10.3389/fphar.2018.01274

**Published:** 2018-11-13

**Authors:** Elia A. Turolla, Silvia Valtorta, Elena Bresciani, Jean-Alain Fehrentz, Liliana Giuliano, Stefano Stucchi, Sara Belloli, Paolo Rainone, Francesco Sudati, Laura Rizzi, Laura Molteni, Pascal Verdiè, Jean Martinez, Antonio Torsello, Rosa Maria Moresco, Sergio Todde

**Affiliations:** ^1^School of Medicine and Surgery, University of Milano-Bicocca, Milan, Italy; ^2^Tecnomed Foundation, University of Milano-Bicocca, Milan, Italy; ^3^National Research Council, Institute of Molecular Bioimaging and Physiology, Milan, Italy; ^4^Institute of Biomolécules Max Mousseron, University of Montpellier, CNRS, ENSCM, School of Pharmacy, Montpellier, France; ^5^Nuclear Medicine Department, San Raffaele Scientific Institute, Milan, Italy; ^6^Doctorate School of Molecular and Translational Medicine, University of Milan, Milan, Italy

**Keywords:** TLQP-21, VGF, obesity, fluorine-18, radiolabeling, PET, biodistribution

## Abstract

TLQP-21 is a neuropeptide that is involved in the control of several physiological functions, including energy homeostasis. Since TLQP-21 could oppose the early phase of diet-induced obesity, it has raised a huge interest, but very little is known about its mechanisms of action on peripheral tissues. Our aim was to investigate TLQP-21 distribution in brain and peripheral tissues after systemic administration using positron emission tomography. We report here the radiolabeling of NODA-methyl phenylacetic acid (MPAA) functionalized JMV5763, a short analog of TLQP-21, with [^18^F]aluminum fluoride. Labeling of JMV5763 was initially performed manually, on a small scale, and then optimized and implemented on a fully automated radiosynthesis system. In the first experiment, mice were injected in the tail vein with [^18^F]JMV5763, and central and peripheral tissues were collected 13, 30, and 60 min after injection. Significant uptake of [^18^F]JMV5763 was found in stomach, intestine, kidney, liver, and adrenal gland. In the CNS, very low uptake values were measured in all tested areas, suggesting that the tracer does not efficiently cross the blood–brain barrier. Pretreatment with non-radioactive JMV5763 caused a significant reduction of tracer uptake only in stomach and intestine. In the second experiment, PET analysis was performed *in vivo* 10–120 min after i.v. [^18^F]JMV5763 administration. Results were consistent with those of the *ex vivo* determinations. PET images showed a progressive increase of [^18^F]JMV5763 uptake in intestine and stomach reaching a peak at 30 min, and decreasing at 120 min. Our results demonstrate that ^18^F-labeling of TLQP-21 analogs is a suitable method to study its distribution in the body.

## Introduction

VGF is a neurohormone involved in the regulation of energy homeostasis (Hahm et al., 1999). It is synthesized as a large pro-peptide and its processing results in a number of biologically active peptides that are expressed throughout the central and the peripheral nervous system and endocrine tissues (Van den Pol et al., 1994; Alder et al., 2003; Levi et al., 2004; Yamaguchi et al., 2007). A 21 aminoacid residues, namely, TLQP-21, is known to regulate glucose response and increases energy expenditure (Bartolomucci et al., 2006). Interestingly, TLQP-21 is stored in secretory vesicles in sympathetic nerve terminals and it is co-secreted with norepinephrine to activate lipolysis (Possenti et al., 2012; Cero et al., 2016). In obese rats, levels of TLQP-21 in brown (BAT) and white (WAT) adipose tissue are differently regulated by modifications in plasma glucose concentration than in lean mice (D’Amato et al., 2015). Indeed, significantly lower levels of TLQP-21 have been reported in plasma and adipose tissue (especially in BAT) of obese mice and in plasma of type 2 diabetes (T2D) patients (D’Amato et al., 2015). Despite many efforts to characterize the physiological effects of TLQP-21, little is known about its molecular targets. The complement 3a receptor 1 (C3aR1) and/or the C1q receptor (gC1qR; Chen et al., 2013; Hannedouche et al., 2013) have been proposed to bind TLQP-21, but this mechanism of action is still matter of debate (Severini et al., 2008). The effects of TLQP-21 have been studied after both central and peripheral administration (Salton et al., 2000; Severini et al., 2009; Possenti et al., 2012); however, its tissue distribution after systemic administration is poorly known, although more information about its kinetic in plasma have been very recently obtained (Guo et al., 2018) by radiolabeling TLQP-21 with the radionuclide iodine-125, which prompt for a fast initial plasma clearance and a significant long-term uptake in adipose tissue. To obtain a better understanding about the *in vivo* kinetics of TLQP-21 and its potential target tissues, we have radiolabeled with fluorine-18 a short analog of TLQP-21, namely, JMV5763, that include the 13 aa residues of the C-terminal region of TLQP-21, and retain the capability to fully stimulate intracellular calcium release in CHO cells (Molteni et al., 2017). The distribution of [^18^F]JMV5763 in brain and peripheral tissues after i.v. administration has been evaluated *ex vivo* and *in vivo* by PET imaging in rat models.

JMV5763 was radiolabeled with [^18^F](AlF)^2+^, which was initially reported as an innovative approach for the peptide labeling with fluorine-18 (McBride et al., 2009). They proposed a simple, versatile method, where fluorine-18 is first reacted with aluminum to form [^18^F](AlF)^2+^. The formation of the AlF complex, and subsequent chelation/conjugation reactions, may occur in aqueous solution, thus eliminating the need for time-consuming drying steps. [^18^F](AlF)^2+^ is then used in combination with suitable chelators, thus indirectly extending to fluorine-18 the potentiality of coordination chemistry. [^18^F](AlF)^2+^ labeled radiotracers proved to be stable, *in vivo* (Dijkgraaf et al., 2012; Selivanova et al., 2012). The radiolabeling procedure with [^18^F](AlF)^2+^ complex has been here tested on a NODA-methyl phenylacetic acid (MPAA) functionalized JMV5763.

Reaction parameters of radiolabeling procedure (concentration, peptide amount, volumes, temperature, etc.) have been initially set following a manual procedure, using low fluorine-18 activity levels, and then scaled up and fully automated using a GE TRACERlab FX-N Pro synthesis module. [^18^F]JMV5763 was obtained in good yield and high purity, and it was then used for biodistribution studies in animal models.

## Materials and Methods

### Materials

Commercially available chemicals and solvents used in the present work were of analytical grade and used without further purification. AlCl_3_⋅6H_2_O 99.9995% and pH 4.0 sodium acetate buffer solutions were purchased from Alfa Aesar (Karlsruhe, Germany). NH_2_-MPAA-NODA chelator was purchased from CheMatech (Dijon, France). TLQP-21 (TLQPPASSRRRHFHHALPPAR), JMV5656 (RRRHFHHALPPAR), and JMV5763 (NODA-βAβARRRHFHHALPPAR) were synthesized by conventional solid phase peptide synthesis and then purified on a C18 reversed phase column; 1 M HCl pH 7.4 phosphate buffered saline (PBS) and trifluoroacetic acid (TFA) were obtained from Sigma Aldrich (St. Louis, MO, United States). Sep-Pak Light Waters Accel Plus QMA were purchased from ABX Chemicals (Radeberg, Germany). SepPak CM, used for fluorine-18 purification, and C18 Plus cartridges, used to purify the radiolabeled peptide, were obtained from Waters (Milford, CT, United States). The analytical reverse-phase HPLC column (Gemini C18 250 mm × 4.6 mm, 5 μm) was bought from Phenomenex (Torrance, CA, United States). Automated radiolabeling tests were carried out using a commercially available automated radiosynthesis module (GE TracerLab FX-N Pro, Uppsala, Sweden).

### Methods

#### Cell Cultures

CHO cells were cultured in HAM’S F12 medium supplemented with 10% heat-inactivated fetal bovine serum (FBS), 100 IU/mL penicillin, 100 μg/mL streptomycin, and 2 mM L-glutamine (Euroclone, Pero, Italy) under standard cell culture conditions (at 37°C, in 5% CO_2_).

#### Intracellular Ca^2+^ Mobilization Assay

CHO cells were plated at 20.000 cells/well into black walled, clear bottom 96-well plate (Corning, Kaiserslautern, Germany) and cultured one day up to 80–90% of confluence. Prior to assay, cells were incubated in dark conditions with 100 μL of Hank’s Balanced Salt Solution (HBSS) containing 20 mM HEPES, 2.5 mM probenecid, and 4.5 μM FLUO-4 NW (Molecular Probes, Eugene, OR, United States) at 37°C and 5% CO_2_ for 45 min. Fluorescence emissions were measured with the multilabel spectrophotometer VICTOR3 (Perkin Elmer, Waltham, MA, United States) at 485/535 nm (excitation/emission filters) every 0.5 s for the 20 s preceding, and the 60 s following the stimulation. TLQP-21, JMV5656, and JMV5763 were diluted in HBSS solution to 10^-6^ M and injected into the wells by an automated injector system.

#### Production of [^18^F]fluoride

[^18^F]fluoride was produced using an 18 MeV proton beam (IBA Cyclone 18/9, Louvain-la-Neuve, Belgium) via the ^18^O(p,n)^18^F nuclear reaction, by irradiation of a niobium target containing 2 mL of >97% enriched [^18^O]water (Rotem, Arava, Israel).

#### Manual Procedure for the Preparation of [^18^F]JMV5763

The cyclotron produced [^18^F]fluoride was loaded onto a QMA cartridge, rinsed with 5 mL of water to remove potential metallic impurities, and eluted with 1 mL of physiological saline solution. The pH of the resulting solution was adjusted with 4.5 μL glacial acetic acid.

**FIGURE 1:**
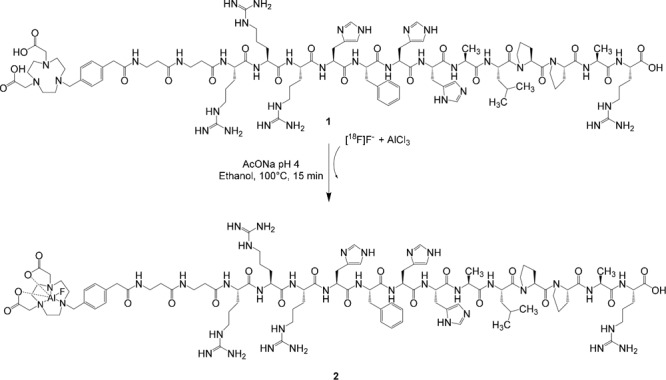
Preparation of [^18^F]JMV5763 **(2)** from [^18^F]fluoride, aluminum chloride, and JMV5763 **(1)** in pH 4.0 sodium acetate buffer.

Five microliters (10 nmol) of 2 mM AlCl_3_⋅6H_2_O stock solution, prepared by dissolving AlCl_3_⋅6H_2_O in a 2 mM pH 4.0 sodium acetate solution, were added to 10 μL (13 nmol) of 1.3 mM JMV5763 stock solution, prepared by adding 1 mg of JMV5763 to 250 μL of 2 mM, pH 4.0 sodium acetate solution; to this solution, 100 μL of EtOH and 100 μL of [^18^F]NaF pH 4.0 (≈180 MBq) were then added. The reaction mixture was heated for 15 min at 100°C. After cooling at room temperature, the mixture was diluted with water (5 mL) and then purified by passing it through a C18 SepPak cartridge previously conditioned with 10 mL of ethanol and 10 mL of water. The cartridge was then washed with 10 mL of water and the product was finally eluted with 1 mL of 10 mM HCl in ethanol (Figure 1).

#### Automated Procedure for the Preparation of [^18^F]JMV5763

The automated preparation of [^18^F]JMV5763 was carried out using a GE TracerlabFx N Pro synthesis module. QMA cartridge was pre-conditioned with 10 mL of saline physiological solution and subsequently dried with air. At the end of cyclotron irradiation, [^18^F]fluoride solution (2.0 mL, ≈6–11 GBq) was collected in a Wheaton vial and passed through the SepPak QMA cartridge as usual. QMA cartridge was then rinsed with 10 mL of water, and [^18^F]fluoride eluted with 0.5 mL of 0.5 M sodium acetate pH 4.0 in the glassy carbon reactor tube, where a mixture of 50 μL (100 nmol) of 2 mM AlCl_3_⋅6H_2_O stock solution and 100 μL (130 nmol) of 1.3 mM JMV5763 stock solution were placed. No pH adjustment was necessary after purified [^18^F]NaF elution; 350 μL of EtOH were then added and the mixture was allowed to react in the sealed reactor at 100°C for 15 min. At the end of reaction, the mixture was cooled to 25°C, diluted with 11.5 mL of water, and passed through a SepPak C18 Plus cartridge (pre-conditioned with 10 mL of EtOH and 10 mL of water). To remove unreacted [^18^F]fluoride, the cartridge was rinsed with 15 mL of water, eluted with 1 mL of 10 mM HCl in EtOH, and finally collected in a vial containing 10 mL saline solution and 100 μL PBS buffer.

#### HPLC Analysis

Both the crude reaction mixtures and purified radiolabeled preparations were analyzed by RP-HPLC on a Perkin Elmer Series 200 system. Analysis conditions were as follows: flow rate: 1 mL/min; mobile phase: 0.1% TFA in water (buffer A) and 0.1% of TFA in acetonitrile (buffer B); gradient: 1 min with 100 % A, then to 90:10 A/B over 2 min, followed by 70:30 A/B over 20 min. Radioactivity of the eluate was monitored using a NaI “flow” radiodetector (Bioscan, Santa Barbara, CA, United States) connected in-line, whereas cold products were detected using an integrated photodiode array detector by setting the absorbance at 220 nm.

#### Stability Testing Phosphate Buffered Saline (PBS)

The purified radiolabeled [^18^F]JMV5763, dissolved in 1 mL of 10 mM HCl in EtOH, was mixed with 10 mL of physiological saline solution and 100 μL of pH 7.4 PBS; different aliquots of this mixture were then analyzed with RP-HPLC at different time intervals (0–60–120–180 min).

#### Animal Studies

Seven to eight weeks old male CD1 mice, weighing 28–30 g (ENVIGO RMS, S. Pietro al Natisone, Italy) were housed under specific pathogen free condition in the animal house facility of San Raffaele Scientific Institute. All procedures involving the animals and their care were conducted in conformity with the related institutional guidelines. Animal experiments were approved by the Ethics Committee of the San Raffaele Scientific Institute and notified to the Italian Ministry of Health.

#### Biodistribution Studies of [^18^F]JMV5763

Mice were injected in the tail vein with 2.86 ± 0.21 MBq of [^18^F]JMV5763 dissolved in 30 μL of saline solution. At 10 (*n* = 8), 30 (*n* = 7), and 60 (*n* = 4) min after injection, mice were sacrificed under deep anesthesia and blood, plasma, heart, lung, liver, spleen, intestine, kidney, stomach, adrenal gland, pancreas, bone, testis, muscle, frontal cortex, striatum, hippocampus, pons, thalamus, hypothalamus, and cerebellum were sampled and washed with cold saline. Tissues were placed in pre-weighed tubes and counted in a gamma counter (LKB Compugamma CS 1282, Mount Waverley, Australia). The radioactivity concentration in tissues was calculated both as percentage of injected dose per gram of tissue (%ID/g) and as organs to blood ratio.

In competition studies, cold JMV5763 (3 mg/kg in vehicle) or vehicle were administered i.v. 15 min before i.v. injection of 2.75 ± 0.22 MBq of [^18^F]JMV5763. Mice were sacrificed 30 min after tracer administration (*n* = 4), tissues were collected as described before and radioactivity measured.

#### PET Imaging Study

Mice underwent 10 min-static PET acquisitions using a YAP-(S)-PET scanner (ISE, Pisa, Italy). In a first group of mice, the acquisition was centered on the head, and in the second group on the trunk. Acquisitions were made at 10, 30, 60, and 120 min after i.v. injection of 3.79 MBq of [^18^F]JMV5763. The YAP-(S)-PET scanner has a field of view (FOV) of 4 cm × 4 cm × 4 cm and it is made up of four detector heads: each one is composed of a 4 cm × 4 cm^2^ YAlO^3^:Ce (or YAP:Ce) matrix of 27 × 27 elements, 1.5 mm × 1.5 mm × 20 mm each, coupled to a R2486 PS-PMT (Hamamatsu, Shizuoka, Japan). The four modules are positioned on a rotating gantry. Mouse was positioned prone on the PET scanner bed and was anesthetized with 2% isoflurane in air during PET acquisition. PET data were acquired in list mode using the full axial acceptance angle of the scanner (3D mode) and then reconstructed with the expectation–maximization (EM) algorithm. All images were calibrated with a dedicated phantom, corrected for the radionuclide half-life decay, and then quantified with PMOD 2.7 software. Regions of interest (ROIs) were drawn on brown adipose tissue for all time points. Radioactivity concentration was reported as maximum standardized uptake value (SUVmax) = radioactivity in the ROI/injected radioactivity × animal weight.

#### Statistical Analysis

Values are expressed as mean ± SEM. The statistical significance of differences between groups was evaluated with the Student’s *t*-test. A *P*-value of less than 0.05 was considered significant.

## Results

### Radiochemistry (Manual Procedure)

**FIGURE 2:**
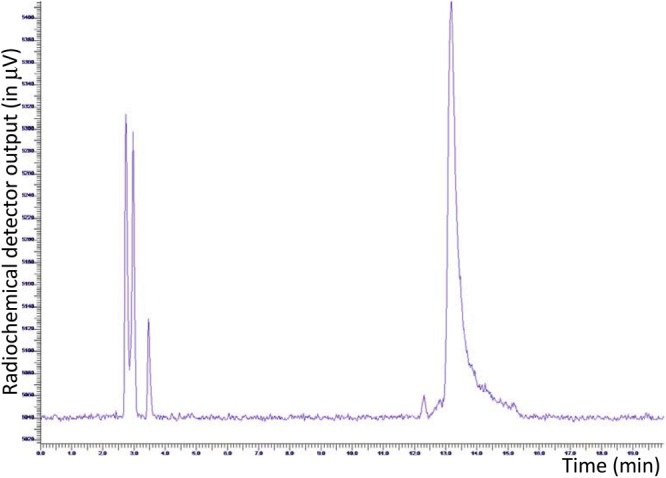
HPLC radio-chromatogram of manually prepared [^18^F]JMV5763 using ethanol as a co-solvent.

As described previously, with the aim to work manually, radiolabeling of JMV5763 was initially performed on a small scale, adapting the procedure originally described by McBride et al. (2009). RP-HPLC analysis of the crude reaction mixture showed one peak due to [^18^F]fluoride (tR 2.9–3.1 min), and a very low intensity peak related to the desired product, which only accounted for about 7% of the total activity. The outcome significantly improved to 16%, when the sodium acetate buffer was replaced with 100 μL of EtOH (D’Souza et al., 2011). To further improve labeling efficiency and to increase volumes in view to adaption to the automated procedure, 50 μL of JMV5763 and 25 μL of aluminum acetate stock solutions were used, keeping constant [^18^F]NaF and ethanol. In these conditions, percentage of fluorine-18 related to [^18^F]JMV5763 improved to 67% (Figure 2).

The effect of temperature on the radiolabeling reaction was also investigated. Labeling yield improved with increasing temperature. Indeed, reaction did not occur at room temperature and up to 50°C, while maximum radiolabeling was obtained at 100°C. The effect of time was also assessed at the temperature of 100°C resulting in an optimal reaction time of 10 min.

### Radiochemistry (Automated Procedure)

One of the drawbacks of the manual procedure was represented by the need to add glacial acetic acid to adjust the pH of the reaction media, which is difficult to handle with automated system, due to the large volume of reagents/solvents reservoirs compared with the necessarily small volume of acetic acid and the need to carefully control the amount added. To overcome this problem, we developed a simple and reliable procedure for purifying and concentrating fluorine-18, using a pre-conditioning step of the QMA cartridge with physiological saline solution and subsequent elution with 0.5 M sodium acetate buffer solution, pH 4.0. Although the elution yield was slightly lower than using saline solution (70 ± 3 vs. 80 ± 5%), this method allowed to automate the procedure.

**FIGURE 3:**
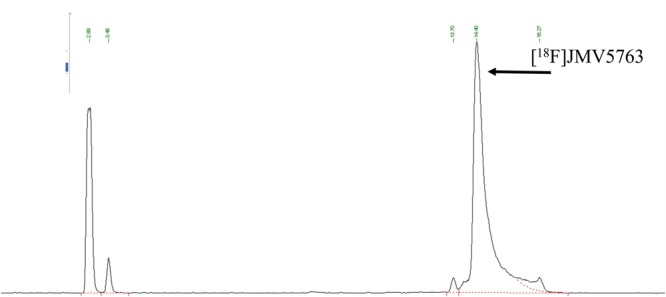
Radio-HPLC chromatogram of the crude reaction mixture for the automated preparation of [^18^F]JMV5763 without purification.

The software sequence was initially developed performing radiolabeling tests with NODA-MPAA chelator alone. The best results were obtained using 500 μL of 0.5 M AcONa to elute [^18^F]fluoride from QMA cartridge, 100 μL of a 2 mM solution of NODA MPAA in 2 mM AcONa pH 4.0, 50 μL of 2 mM AlCl_3_ stock solution, and 150 μL ethanol; reaction temperature was set to 100°C and reaction time to 15 min. Radio-HPLC analysis of the reaction mixture showed a peak area percentage of 54% for the desired radiolabeled chelator, 44% due to unreacted [^18^F]fluoride, and 1% of radiochemical impurities. Taking profit of the above test, radiolabeling conditions for the reaction were optimized as follows: 100 μL of peptide stock solution, 50 μL of aluminum chloride stock solution, 500 μL of 0.5 M AcONa pH 4.0, and 350 μL ethanol. The total synthesis time necessary to obtain purified and formulated [^18^F]JMV5763 was about 30 min. RP-HPLC of the crude reaction mixture showed that about 70% of the activity was attributable to the radiolabeled peptide (Figure 3).

**FIGURE 4:**
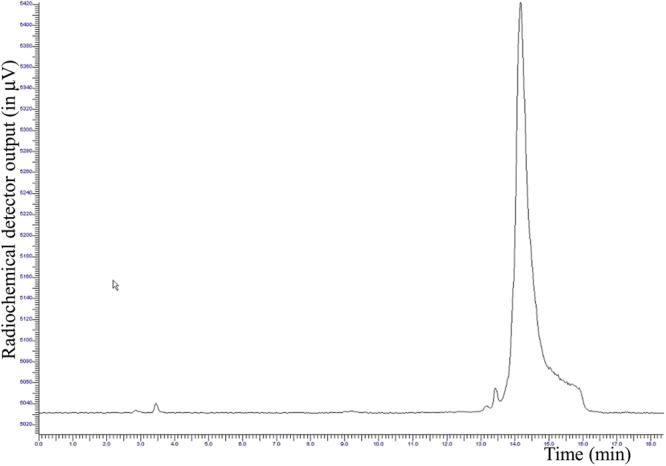
Radio-HPLC chromatogram of the purified [^18^F]JMV5763 (t_R_∼14.5 min, purity 99.0 ± 5%).

After SepPak C18 plus purification, the radiochemical purity (RCP) was in the range of 99.0 ± 5% (Figure 4).

**FIGURE 5:**
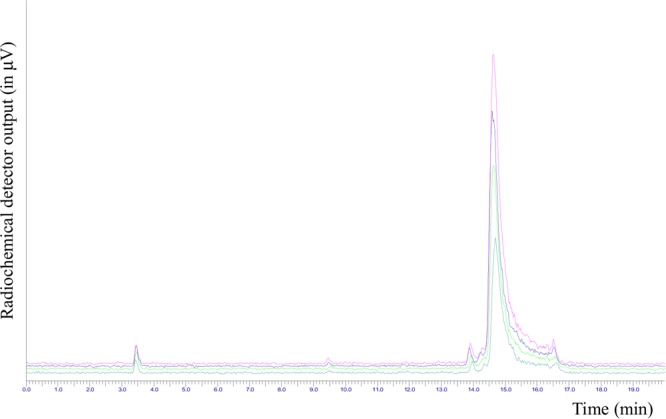
Overlapping of the radio-HPLC chromatograms of [^18^F]JMV5763 in PBS solution to evaluate stability at different time intervals.

Radiochemical yield, not corrected for decay, was of 33.8 ± 2%. Stability was tested in 10 mL of physiological saline solution and 100 μL of PBS, pH 7.4, up to 3 h at room temperature, and analyzing aliquots at 60 min time intervals; radio-HPLC analysis showed that the tracer was stable in the above conditions, and no significant release of [^18^F]AlF or other fluorine-18 labeled impurities were observed, with RCP ranging from 98.5 to 97.7% (Figure 5).

### Intracellular Calcium Stimulation

**FIGURE 6:**
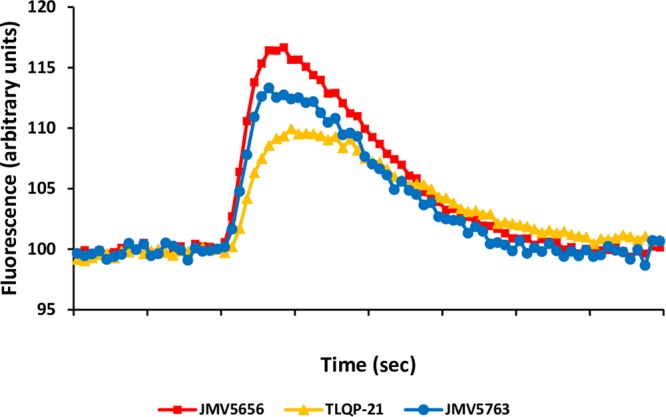
JMV5763 stimulation of intracellular Ca^2+^ levels in CHO cells. JMV5763, JMV5656, and TLQP-21 were used at 10^-6^ M. Cells were loaded with FLUO-4 NW and fluorescence emissions were measured at 485/535 nm (excitation/emission filters) every 0.5 s for the 60 s following injection of the stimuli. Data are the mean of six determinations for each point.

The effects of TLQP-21, JMV5656, and JMV5763 were tested *in vitro* in CHO cells. JMV5763 (1 μM) stimulated a significant increase (*P* < 0.05) in intracellular Ca^2+^ levels in CHO cells. Its effect was comparable to those of TLQP-21 and its shorter synthetic analog, JMV5656. These results demonstrate that JMV5763 has retained the capability to stimulate its binding sites on CHO cells and it is suitable for further studies (Figure 6).

### Time-Course of Tissue Distribution of [^18^F]JMV5763

[^18^F]JMV5763 was administered i.v. in healthy CD1 mice. The kinetic of distribution in samples of interest was performed at 10 (*n* = 8), 30 (*n* = 7), and 60 min (*n* = 4) post-injection. Uptake values have been calculated as percentage of injected dose per gram of tissue (%ID/g; Table 1) or as sample to blood ratio (%ID/g/%ID/g; Table 2). In peripheral tissues, a high uptake of [^18^F]JMV5763 was found in stomach, intestine, kidney, liver, and adrenal gland (Table 1). Interestingly, the above tissues have been proposed as functional sites for TLQP-21 and for the expression of its binding sites (Possenti et al., 2012; Lewis et al., 2015). In these tissues, radioactivity (%ID/g) reached maximum concentration after 10 min, remained steady at 30 min, and decreased at 60 min. The radiotracer levels were low in all the other considered tissues (Table 1).

**Table 1:** Time-course of [^18^F]JMV5763 tissue distribution after i.v. administration.

Tissue	At 10 min	At 30 min	At 60 min	Competitor pre-administration at 30 min
	(*n* = 8)	(*n* = 7)	(*n* = 4)	(*n* = 4)
**(%ID/g)**				
Blood	4.12 ± 1.41	1.84 ± 0.87	0.41 ± 0.12	2.76 ± 0.94
Plasma	7.95 ± 2.19	3.07 ± 1.54	0.67 ± 0.25	4.63 ± 1.46
Heart	1.84 ± 0.56	0.75 ± 0.21	0.30 ± 0.15	1.14 ± 0.42
Lung	2.79 ± 0.67	1.66 ± 1.12	0.35 ± 0.17	1.82 ± 0.56
Liver	1.73 ± 0.48	1.99 ± 0.72	1.84 ± 0.42	1.82 ± 0.70
Spleen	0.97 ± 0.22	0.52 ± 0.24	0.18 ± 0.05	0.76 ± 0.37
Intestine	2.72 ± 1.56	1.59 ± 0.55	1.21 ± 0.80	1.02 ± 0.47
Kidney	12.05 ± 2.77	8.97 ± 2.51	5.31 ± 1.40	11.32 ± 6.54
Stomach	2.49 ± 1.15	1.25 ± 0.18	0.36 ± 0.17	0.84 ± 0.16^∗^
Adrenal	3.13 ± 1.47	3.10 ± 2.93	0.35 ± 0.24	2.72 ± 1.13
Pancreas	1.37 ± 0.66	0.63 ± 0.27	0.16 ± 0.06	0.73 ± 0.27
Bone	1.22 ± 0.58	0.96 ± 0.53	0.47 ± 0.05	0.97 ± 0.30
Testicles	0.74 ± 0.24	0.35 ± 0.18	0.09 ± 0.01	0.50 ± 0.11
Muscle	1.57 ± 0.41	0.61 ± 0.22	0.15 ± 0.02	0.92 ± 0.38
Cortex	0.13 ± 0.05	0.06 ± 0.03	0.02 ± 0.01	0.08 ± 0.04
Striate	0.09 ± 0.03	0.05 ± 0.05	0.01 ± 0.00	0.04 ± 0.02
Hyppocampus	0.12 ± 0.04	0.08 ± 0.07	0.02 ± 0.01	0.06 ± 0.02
Pons	0.23 ± 0.12	0.12 ± 0.13	0.02 ± 0.01	0.10 ± 0.05
Thalamus	0.11 ± 0.04	0.10 ± 0.12	0.01 ± 0.00	0.08 ± 0.04
Hypothalamus	0.13 ± 0.03	0.08 ± 0.07	0.01 ± 0.00	0.08 ± 0.02
Cerebellum	0.13 ± 0.03	0.07 ± 0.04	0.02 ± 0.00	0.09 ± 0.04

*The radiotracer was injected in the tail vein and mice were sacrificed after 10, 30, and 60 min. Radioactivity concentrations are expressed as percentage of injected dose per gram of tissue (%ID/g). The specificity of radiotracer accumulation was confirmed by competition experiments with unlabeled JMV5763 administration. Values are expressed as mean ± SD (^∗^*P* < 0.05 vs. corresponding 30 min value).*

By calculating the radioactivity in terms of samples to blood ratio (%ID/g/%ID/g), a progressive time-dependent increase appeared in intestine, bone, stomach, heart, liver, and kidney (Table 2).

**Table 2:** Time-course of [^18^F]JMV5763 tissue/blood distribution after i.v. administration.

Tissue to blood ratio	At 10 min	At 30 min	At 60 min	Competitor pre-administration at 30 min
	(*n* = 8)	(*n* = 7)	(*n* = 4)	(*n* = 4)
**(%ID/g/%ID/g)**				
Plasma	2.04 ± 0.70	1.71 ± 0.09	1.60 ± 0.23	1.69 ± 0.06
Heart	0.49 ± 0.24	0.53 ± 0.32	0.82 ± 0.51	0.43 ± 0.19
Lung	0.79 ± 0.24	0.90 ± 0.31	0.85 ± 0.22	0.67 ± 0.03
Liver	0.51 ± 0.23	1.32 ± 0.67	4.68 ± 1.40	0.70 ± 0.33
Spleen	0.25 ± 0.08	0.32 ± 0.15	0.46 ± 0.16	0.27 ± 0.07
Intestine	0.70 ± 0.37	1.19 ± 0.91	3.04 ± 1.85	0.36 ± 0.07^∗^
Kidney	3.17 ± 0.90	5.58 ± 1.91	13.08 ± 1.92	4.23 ± 2.30
Stomach	0.65 ± 0.32	0.90 ± 0.61	0.90 ± 0.36	0.36 ± 0.08^∗^
Adrenal	0.78 ± 0.35	1.70 ± 1.50	1.11 ± 0.97	1.00 ± 0.41
Pancreas	0.33 ± 0.11	0.39 ± 0.18	0.40 ± 0.15	0.28 ± 0.12
Bone	0.31 ± 0.12	0.58 ± 0.22	1.37 ± 0.43	0.37 ± 0.07
Testicles	0.20 ± 0.07	0.22 ± 0.09	0.26 ± 0.07	0.20 ± 0.07
Muscle	0.40 ± 0.12	0.43 ± 0.12	0.43 ± 0.16	0.33 ± 0.08
Cortex	0.03 ± 0.02	0.03 ± 0.01	0.05 ± 0.01	0.03 ± 0.01
Striate	0.02 ± 0.01	0.03 ± 0.02	0.02 ± 0.01	0.02 ± 0.00
Hyppocampus	0.03 ± 0.01	0.04 ± 0.03	0.05 ± 0.02	0.02 ± 0.00
Pons	0.06 ± 0.03	0.07 ± 0.06	0.05 ± 0.01	0.04 ± 0.01
Thalamus	0.03 ± 0.01	0.05 ± 0.06	0.04 ± 0.01	0.03 ± 0.01
Hypothalamus	0.03 ± 0.01	0.04 ± 0.02	0.03 ± 0.02	0.03 ± 000
Cerebellum	0.03 ± 0.01	0.04 ± 0.02	0.05 ± 0.02	0.03 ± 0.00

*Radioactivity concentrations are expressed as tissue to blood ratio (%ID/g/%ID/g). The specificity of radiotracer accumulation was confirmed by competition experiments with unlabeled JMV5763 administration. Values are expressed as mean ± SD (^∗^*P* < 0.05 vs. corresponding 30 min value).*

Pretreatment with cold JMV5763 caused a significantly lower uptake in stomach and intestine with an inhibition of 60 and 70%, respectively (*P* < 0.05; Table 2). In CNS areas, the uptake of the tracer after pretreatment with cold JMV5763 did not change significantly (Tables 1, 2).

In the CNS, low uptake values reached a maximum at 10 min in all tested areas, and thereafter values decreased over time. T/B ratios were low and steady over time indicating that [^18^F]JMV5763 did not efficiently cross the blood–brain barrier (Table 2).

### PET Imaging Study

**FIGURE 7:**
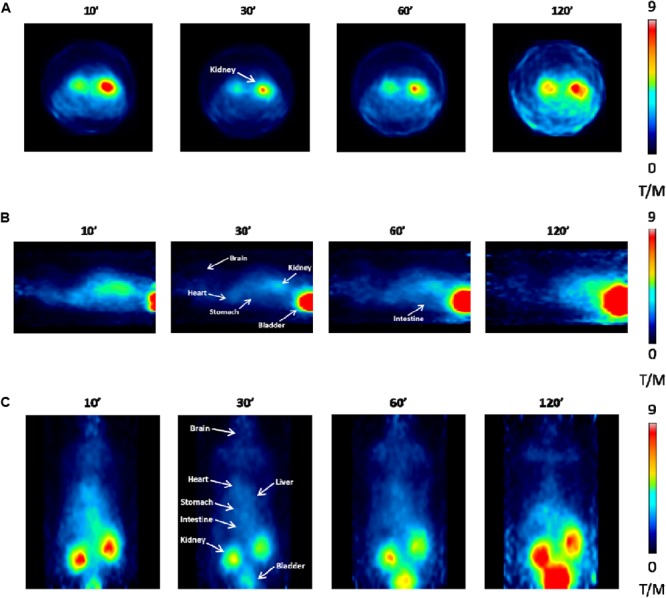
PET images performed at different times (10, 30, 60, and 120 min) after [^18^F]JMV5763 injection. **(A)** Transaxial view. **(B)** Sagittal view. **(C)** Coronal view. White arrows indicate the main areas of interest. Color scale is expressed as tissue to muscle ratio.

PET analysis performed *in vivo* at 10, 30, 60, and 120 min post [^18^F]JMV5763 injection was consistent with the findings obtained from *ex vivo* experiments. Indeed, in CNS areas, the uptake of the tracer was very low and did not change over time (Figure 7). In peripheral tissues, images showed a progressive increase of [^18^F]JMV5763 uptake in specific districts such as intestine and stomach, reaching a peak at 30 min, then remaining steady up to 60 min, and decreasing at 120 min. In the kidney and the bladder, the uptake increased with time. Interestingly, in the brown adipose tissue, SUV was 0.111 at 10 min, increased at 30 min (0.179), and remained steady up to 60 min (0.164), returning to basal values (0.090) at 120 min (data not shown) (Figure 7).

## Discussion

In the present paper, we report for the first time the peripheral tissue distribution of an analog of TLQP-21, namely, JMV5763, after i.v. administration in mice. To this aim, we developed a method for radiolabeling of JMV5763 with fluorine-18. This radiotracer allowed to evaluate the peptide distribution using *ex vivo* and *in vivo* emission tomography techniques. Moreover, a secondary objective of our study was to explore a potential use of the radiolabeled peptide as potential imaging probe.

VGF is a neurohormone with potential links with obesity. In fact, VGF-derived peptides are upregulated in the hypothalamus of rats fed with a high-fat hypercaloric diet (Chakraborty et al., 2006). TLQP-21 is an internal C-terminal fragment of VGF, whose expression was characterized in various endocrine and neuronal cells, in the gastrointestinal tract and adipose tissue (Levi et al., 2004). Very little results supporting a peripheral role for TLQP-21 have been published, to date. In fact, most findings on TLQP-21 effects derive from its intraventricular administration in the brain, but recently a chronic peripheral TLQP-21 administration (i.p.) proved to be effective in reducing body weight and fat mass in obese mice (Bartolomucci et al., 2006). Consistently, a high-affinity binding site for TLQP-21 has been characterized in adipocyte membranes. The existence of other peripheral target tissues of TLQP-21 has been proposed but never investigated systematically. To establish whether TLQP-21 has a preferential distribution in the body, we explored the use of a new radiolabeled probe for non-invasive monitoring (PET imaging). In addition, considering the potential interest of TLQP-21 in the study of obesity, secondary objective of our study was to explore a potential use of the radiolabeled peptide as potential imaging probe.

Due to its favorable characteristics of energy emission, half-life, ease, and yield of production, fluorine-18 is the most used PET radionuclide for radiolabeling small molecules. Its direct insertion in the structure of “biological” molecules, such as peptides, nucleic acids, antibodies, etc., has long been limited by the need to radiolabel prostethic groups such as N-succinimidyl-4-[^18^F]fluorobenzoate ([^18^F]SFB) or [^18^F]fluorobenzaldehyde, and their subsequent conjugation to the biomolecule (Richter and Wuest, 2014). These methods suffer of major drawbacks, since they are multi-step, time-consuming radiosynthesis (up to 2–3 h), which often proceed with poor radiochemical yields and pose significant challenges for their automation. Novel and more efficient methods have been more recently introduced, like the use of [^18^F](AlF)^2+^ complexes, that enables an easy and direct access to radiofluorination of peptides and proteins conjugated with suitable bi-functional metal chelators and allows to label these molecules in a single step and under milder conditions. Among the available metal chelators, 1,4,7-triazacyclononane-diacetic acid (NODA) has been shown to possess excellent binding kinetics toward [^18^F](AlF)^2+^ complexes, especially when attached to a MPAA group, achieving highly stable *in vivo* complexes (D’Souza et al., 2011). These radiolabeling procedures are mainly performed manually, using small reagents/solvents volumes and low amount of peptides. In the present study, we report the fluorine-18 radiolabeling of NODA-MPAA functionalized JMV5763, a short analog of TLQP-21, via automated radiosynthesis method; with a starting activity of 20 GBq of [^18^F]fluoride, 5.6 GBq of final purified product could be obtained. RP-HPLC analysis of the crude reaction mixture showed that about 70% of the activity was bound to the peptide, as determined by peak area percentage. The final method can be performed in one pot, is sufficiently fast (30 min) and, after purification through solid phase C18 plus cartridge, allows to obtain radiolabeled [^18^F]JMV5763 in a satisfactory yield (RCY = 33.8 ± 2%) and high purity (RCP = 99.0 ± 5%), which are suitable for further pre-clinical studies. This approach has the advantage to reduce personnel radiation exposure, as well as to obtain a reproducible method easily adaptable to the radiolabeling of a wide variety of biological molecules of potential interest.

Our *ex vivo* and *in vivo* tissue distribution results suggest that after i.v. injection, [^18^F]JMV5763 initially accumulated in regions expressing TLQP-21 binding sites such as stomach, intestine, and adrenal gland, but also in lungs. In brain regions the radioactivity remained very low at all times, similarly to results reported with iodinated TLQP-21 (Guo et al., 2018). This suggests low penetrability of [^18^F]JMV5763 through the BBB, or a very rapid washout from this district. After the initial distribution, [^18^F]JMV5763 was rapidly cleared from all districts, except for liver and kidney. Administration of a loading dose of unlabeled peptide caused a slight decrease of the radiolabeled probe only in stomach and intestine. These findings are consistent with the reported expression of TLQP-21 specific binding sites in intestine and stomach and the whole body organ distribution reported for [^125^I]TLQP-21 (Guo et al., 2018).

## Conclusion

We report here the original characterization of the tissue distribution of a fluorine-18 labeled analog of the peptide TLQP-21. After intravenous administration, our findings demonstrate that only in the stomach and intestine it is possible to measure a specific low binding of the tracer, whereas no other peripheral tissues appear to accumulate [^18^F]JMV5763. Despite the potential effect of [^18^F]AlF fluorination on the kinetics of distribution of JMV5763, these results provide a basis for the known biological activity of TLQP-21 in the gastrointestinal tract. We did not measure the accumulation of [^18^F]JMV5763 in the white adipose tissue, but our findings suggest a possible specific binding on the brown adipose tissue.

Furthermore, this study establishes that the NODA-MPAA functionalization of peptides, and their subsequent radiolabeling with [^18^F](AlF)^2+^, provides a potentially useful tool for the *in vivo* study of biological functions. Finally, the proposed automated radiosynthesis method could be easily adapted to other automated equipment, thus preventing personnel exposure to radiation.

## Author Contributions

ET designed the radiolabeling procedure. LG, SS, and FS performed radiosynthesis tests. SB and SV designed biodistribution and PET imaging studies. PR was involved in data analysis, preclinical study design, and manuscript drafting. EB, LR, and LM performed *in vitro* experiments and the calcium assay and analyzed the data. AT, ST, and RM planned the study, analyzed the data, and wrote the final manuscript. J-AF, PV, and JM synthesized TLQP-21 analogs and revised the manuscript.

## Conflict of Interest Statement

The authors declare that the research was conducted in the absence of any commercial or financial relationships that could be construed as a potential conflict of interest.

## References

[B1] Alder J. Thakker-Varia S. Bangasser D. A. Kuroiwa M. Plummer M. R. Shors T. J. (2003). Brain-derived neurotrophic factor-induced gene expression reveals novel actions of VGF in hippocampal synaptic plasticity. *J. Neurosci.* 23 10800–10808. 10.1523/JNEUROSCI.23-34-10800.2003 14645472 PMC3374594

[B2] Bartolomucci A. La Corte G. Possenti R. Locatelli V. Rigamonti A. E. Torsello A. (2006). TLQP-21, a VGF-derived peptide, increases energy expenditure and prevents the early phase of diet-induced obesity. *Proc. Natl. Acad. Sci. U.S.A.* 103 14584–14589. 10.1073/pnas.0606102103 16983076 PMC1600003

[B3] Cero C. Razzoli M. Han R. Sahu B. S. Patricelli J. Guo Z. (2016). The neuropeptide TLQP-21 opposes obesity via C3aR1-mediated enhancement of adrenergic-induced lipolysis. *Mol. Metab.* 6 148–158. 10.1016/j.molmet.2016.10.005 28123945 PMC5220279

[B4] Chakraborty T. R. Tkalych O. Nanno D. Garcia A. L. Devi L. A. Salton S. R. (2006). Quantification of VGF- and pro-SAAS-derived peptides in endocrine tissues and the brain, and their regulation by diet and cold stress. *Brain Res.* 1089 21–32. 10.1016/j.brainres.2006.02.124 16631141

[B5] Chen Y. C. Pristera A. Ayub M. Swanwick R. S. Karu K. Hamada Y. (2013). Identification of a receptor for neuropeptide VGF and its role in neuropathic pain. *J. Biol. Chem.* 288 34638–34646. 10.1074/jbc.M113.510917 24106277 PMC3843076

[B6] D’Amato F. Noli B. Angioni L. Cossu E. Incani M. Messana I. (2015). VGF peptide profiles in type 2 diabetic patients’ plasma and in obese mice. *PLoS One* 10:e0142333. 10.1371/journal.pone.0142333 26562304 PMC4643017

[B7] Dijkgraaf I. Franssen G. M. McBride W. J. D’Souza C. A. Laverman P. Smith C. J. (2012). PET of tumors expressing gastrin-releasing peptide receptor with an 18-F labeled bombesin. *J. Nucl. Med.* 53 947–952. 10.2967/jnumed.111.100891 22570329

[B8] D’Souza C. A. McBride W. J. Sharkey R. M. Todaro L. J. Goldenberg D. M. (2011). High-yielding aqueous 18F-labeling of peptides via Al18F chelation. *Bioconjug. Chem.* 22:1793. 10.1021/bc200175c 21805975 PMC3178738

[B9] Guo Z. Sahu B. S. He R. Finan B. Cero C. Verardi R. (2018). Clearance kinetics of the VGF-derived neuropeptide TLQP-21. *Neuropeptides* 71 97–103. 10.1016/j.npep.2018.06.003 29958697 PMC6166661

[B10] Hahm S. Mizuno T. M. Wu T. J. Wisor J. P. Priest C. A. Kozak C. A. (1999). Targeted deletion of the Vgf gene indicates that the encoded secretory peptide precursor plays a novel role in the regulation of energy balance. *Neuron* 23 537–548. 10.1016/S0896-6273(00)80806-5 10433265

[B11] Hannedouche S. Beck V. Leighton-Davies J. Beibel M. Roma G. Oakeley E. J. (2013). Identification of the C3a receptor (C3AR1) as the target of the VGF-derived peptide TLQP-21 in rodent cells. *J. Biol. Chem.* 288 27434–27443. 10.1074/jbc.M113.497214 23940034 PMC3779738

[B12] Levi A. Ferri G. L. Watson E. Possenti R. Salton S. R. (2004). Processing, distribution, and function of VGF, a neuronal and endocrine peptide precursor. *Cell. Mol. Neurobiol.* 24 517–533. 10.1023/B:CEMN.0000023627.79947.22 15233376 PMC11529936

[B13] Lewis J. E. Brameld J. M. Jethwa P. H. (2015). Neuroendocrine role for VGF. *Front. Endocrinol.* 6:3 10.3389/fendo.2015.00003 PMC4313783 25699015

[B14] McBride W. J. Sharkey R. M. Karacay H. D’Souza C. A. Rossi E. A. Laverman P. (2009). A novel method of 18F radiolabeling for PET. *J. Nucl. Med.* 50:991. 10.2967/jnumed.108.060418 19443594

[B15] Molteni L. Rizzi L. Bresciani E. Possenti R. Petrocchi Passeri P. Ghè C. (2017). Pharmacological and biochemical characterization of TLQP-21 activation of a binding site on CHO cells. *Front. Pharmacol.* 8:167. 10.3389/fphar.2017.00167 28424618 PMC5371653

[B16] Possenti R. Muccioli G. Petrocchi P. Cero C. Cabassi A. Vulchanova L. (2012). Characterization of a novel peripheral pro-lipolytic mechanism in mice: role of VGF-derived peptide TLQP-21. *Biochem. J.* 441 511–522. 10.1042/BJ20111165 21880012

[B17] Richter S. Wuest F. (2014). 18F-labeled peptides: the future is bright. *Molecules* 19 20536–20556. 10.3390/molecules191220536 25493636 PMC6271677

[B18] Salton S. R. Ferri G. L. Hahm S. Snyder S. E. Wilson A. J. Possenti R. (2000). VGF: a novel role for this neuronal and neuroendocrine polypeptide in the regulation of energy balance. *Front. Neuroendocrinol.* 21:199–219. 10.1006/frne.2000.0199 10882540

[B19] Selivanova S. Muller A. Kramer S. Schibli R. Ametamey S. Graham K. (2012). Radiosynthesis and pre-clinical evaluation of Al18F bombesin antagonist: promising tracer for prostate cancer imaging. *J. Nucl. Med.* 53:1732.

[B20] Severini C. Ciotti M. T. Biondini L. Quaresima S. Rinaldi A. M. Levi A. (2008). TLQP-21, a neuroendocrine VGF-derived peptide, prevents cerebellar granule cells death induced by serum and potassium deprivation. *J. Neurochem.* 104 534–544. 10.1111/j.1471-4159.2007.05068.x 18173805

[B21] Severini C. La Corte G. Improta G. Broccardo M. Agostini S. Petrella C. (2009). In vitro and in vivo pharmacological role of TLQP-21, a VGF-derived peptide, in the regulation of rat gastric motor functions. *Br. J. Pharmacol.* 157 984–993. 10.1111/j.1476-5381.2009.00192.x 19466987 PMC2737657

[B22] Van den Pol A. N. Bina K. Decavel C. Ghosh P. (1994). VGF expression in the brain. *J. Comp. Neurol.* 347 455–469. 10.1002/cne.903470311 7822494

[B23] Yamaguchi H. Sasaki K. Satomi Y. Shimbara T. Kageyama H. Mondal M. S. (2007). Peptidomic identification and biological validation of neuroendocrine regulatory peptide-1 and -2. *J. Biol. Chem.* 282 26354–26360. 10.1074/jbc.M701665200 17609209

